# New Insights into the Hypotensins from *Tityus serrulatus* Venom: Pro-Inflammatory and Vasopeptidases Modulation Activities

**DOI:** 10.3390/toxins13120846

**Published:** 2021-11-26

**Authors:** Bruno Duzzi, Cristiane Castilho Fernandes Silva, Roberto Tadashi Kodama, Daniela Cajado-Carvalho, Carla Cristina Squaiella-Baptistão, Fernanda Calheta Vieira Portaro

**Affiliations:** 1 Laboratory of Structure and Function of Biomolecules, Butantan Institute, São Paulo 05503-900, SP, Brazil; cristiane.silva@esib.butantan.gov.br (C.C.F.S.); pararoberval@gmail.com (R.T.K.); daniela.carvalho@butantan.gov.br (D.C.-C.); 2 Immunochemistry Laboratory, Butantan Institute, São Paulo 05503-900, SP, Brazil; carla.baptistao@butantan.gov.br

**Keywords:** *Tityus serrulatus*, venom components, hypotensins, NEP inhibition, cytokines

## Abstract

The *Tityus serrulatus* scorpion is considered the most dangerous of the Brazilian fauna due to the severe clinical manifestations in injured victims. Despite being abundant components of the venom, few linear peptides have been characterized so far, such as hypotensins. In vivo studies have demonstrated that hypotensin I (TsHpt-I) exerts hypotensive activity, with an angiotensin-converting enzyme (ACE)-independent mechanism of action. Since experiments have not yet been carried out to analyze the direct interaction of hypotensins with ACE, and to deepen the knowledge about these peptides, hypotensins I and II (TsHpt-II) were studied regarding their modulatory action over the activities of ACE and neprilysin (NEP), which are the peptidases involved in blood pressure control. Aiming to search for indications of possible pro-inflammatory action, hypotensins were also analyzed for their role in murine macrophage viability, the release of interleukins and phagocytic activity. TsHpt-I and -II were used in kinetic studies with the metallopeptidases ACE and NEP, and both hypotensins were able to increase the activity of ACE. TsHpt-I presented itself as an inhibitor of NEP, whereas TsHpt-II showed weak inhibition of the enzyme. The mechanism of inhibition of TsHpt-I in relation to NEP was defined as non-competitive, with an inhibition constant (Ki) of 4.35 μM. Concerning the analysis of cell viability and modulation of interleukin levels and phagocytic activity, BALB/c mice’s naïve macrophages were used, and an increase in TNF production in the presence of TsHpt-I and -II was observed, as well as an increase in IL-6 production in the presence of TsHpt-II only. Both hypotensins were able to increase the phagocytic activity of murine macrophages in vitro. The difference between TsHpt-I and -II is the residue at position 15, with a glutamine in TsHpt-I and a glutamic acid in TsHpt-II. Despite this, kinetic analyzes and cell assays indicated different actions of TsHpt-I and -II. Taken together, these results suggest a new mechanism for the hypotensive effects of TsHpt-I and -II. Furthermore, the release of some interleukins also suggests a role for these peptides in the venom inflammatory response. Even though these molecules have been well studied, the present results suggest a new mechanism for the hypotensive effects of TsHpt-I

## 1. Introduction

The image of the scorpion has long been connected to human history, being represented in cults, legends, philosophy and arts, as it is one of the oldest animals on the planet. Dating from the Silurian period, more than 400 million years ago, scorpions are organisms that have long intrigued human beings [1,2].

The order Scorpiones is represented by 2200 species and, through taxonomic studies, have been grouped into 20 families and 165 genera of scorpions. The most dangerous, and capable of causing fatal accidents in humans, belong to the Buthidae family, represented by the following genera: *Androctonus* and *Leiurus* (North Africa and Middle East), *Centruroides* (Mexico and the United States) and *Tityus* (South America and Trinidad) [3].

In Brazil, the scorpions *Tityus serrulatus*, *T. bahiensis* and *T. stigmurus* are the animals responsible for serious accidents. Among these, the *T. serrulatus* scorpion, popularly known as the “Brazilian yellow scorpion”, is the one with the highest Medical and Scientific relevance in Brazil. It is mainly distributed among the states of Bahia, Goiás (including the Federal District), Paraná, Espírito Santo, Rio de Janeiro, Minas Gerais and São Paulo [4].

*T. serrulatus* reproduces by parthenogenesis, and each female is able to generate about 70 offspring during its life. They are commonly found in sewers, cemeteries and wastelands, where they find safe shelter and plenty of food. Therefore, in addition to the potency of its venom, their adaptation to urban centers may explain the significant increase in the number of accidents caused by this scorpion in Brazil [4,5,6].

In this scenario, accidents caused by *T. serrulatus* stings are considered a public health problem in Brazil due to its potential to cause severe clinical manifestations, which might bring a prognosis of death, especially on children aged from 0 to 14 years. Although most of the cases have been classified as mild, the biggest concern is related to the high number of cases that are reported annually in Brazil, since scorpion stings represent 41% of all venomous animal accidents, including snakes, spiders, bees and others, as reported in 2016 [7].

Generally, the *T. serrulatus* venom (TsV) is composed of mucus, inorganic salts, lipids, amines, nucleotides, enzymes, kallikrein inhibitors, natriuretic peptides, high molecular weight proteins, peptides, amino acids and neurotoxins [8]. Current studies carried out by Oliveira and colleagues [9] involving the transcriptome of the venom glands have shown that more than 30% of the venom is made up of enzymes and, approximately, 40% of peptides. The peptides present in the TsV can be classified as structured—which are stabilized by disulfide bonds—or linear [8]. The so-called structured peptides have been the most studied components, classified as neurotoxins that interact with ion channels (Na^+^ and K^+^) and are related to the most serious effects caused by the venom. On the other hand, linear peptides, although found with some abundance in the venom, are still poorly characterized. The peptidomic analysis performed by Rates and colleagues [10] demonstrated the existence of a great diversity of peptides in the TsV, all of them not yet characterized. Many could not be found in the database, and as this information is scarce, this requires de novo sequencing. Other studies using “omic” techniques have demonstrated the complexity of the venom in relation to linear peptides from post-translational modifications of larger proteins, generating lists with hundreds of components [11,12,13].

Among the linear peptides we have the hypotensins (TsHpt), identified from TsV proteomic analyzes. Both peptides are made up of 25 amino acid residues that contain two consecutive prolines in their C-terminal portion and a punctual difference between TsHpt-I and -II, which is the residue at position 15, being a glutamine in TsHpt-I and a glutamic acid in TsHpt-II. Studies with TsHpt-I, also known as Ts14, showed that this peptide was able to exert hypotensive activity in normotensive Wistar rats by potentiating bradykinin. The hypothesis is that the vasodilation effect is related to the release of NO by an independent mechanism of ACE inhibition [14].

In order to increase knowledge about hypotensins and their biological activities, the present work demonstrates, for the first time, the interaction of these peptides with human vasopeptidases, ACE (EC 3.4.15.1) and NEP (EC 3.4.24.11), alongside with cellular assays, which were carried out in order to verify the possible action of hypotensins as inflammatory or anti-inflammatory peptides.

## 2. Results

### 2.1. Modulation of ACE and NEP Activities by Hypotensins

For the initial kinetic tests, the synthetic hypotensins were incubated with the metallopeptidases ACE and NEP and their fluorescent substrates, Abz-FRK(Dnp)P-OH and Abz-RGFK (Dnp)-OH, respectively. These enzymes were chosen for the studies because they are considered of medical importance, where their modulations caused by venom peptides may be related to some symptoms present in serious accidents, such as hypotension/hypertension. As shown in Table 1, the hypotensins showed different activities in relation to the modulations of the peptidases studied.

The results were obtained by incubating the enzymes with their FRET substrates (10 µM) in a final volume of 100 µL. For the ACE assays, the used buffer was Tris HCl 100 mM, NaCl 50 mM and ZnCl_2_ 10 µM, pH 7.0. For NEP, the assays were made in Tris HCl 50 mM, pH 7.5 buffer. All reactions occurred at 37 °C, in a Victor 3 fluorimeter (Perkin–Elmer) adjusted for excitation and emission readings at 320 and 420 nm, respectively, for 15 min (one reader per minute). Results were obtained in triplicate and analyzed on GraFit 5 software.

Both peptides increased the catalytic activity of ACE, and TsHpt-I was more effective than TsHpt-II in activating the enzyme. Contrary to that observed with ACE, the results involving the peptides tested with NEP revealed the presence of inhibitors of this enzyme. As a highlight, TsHpt-I was able to reduce by 75% the hydrolysis of the FRET substrate used.

As already mentioned, the suggested mechanism of action on hypotension caused by TsHpt-I in vivo is the release of NO, together with the agonistic effect on bradykinin B2 receptors [15]. Taken together, our results with TsHpt-I seem to indicate a second hypotensive mechanism for this peptide, which involves the inhibition of NEP. Interestingly, TsHpt-I showed a strong inhibition of NEP’s catalytic activity, in contrast with TsHpt-II. This fact is probably the result of a single difference between the primary structures between the hypotensins; that is, the presence of glutamine at position 15 in TsHpt-I, instead of a glutamic acid present in TsHpt-II.

### 2.2. Stability of Hypotensins against Metallopeptidases ACE and NEP

As hypotensins showed interactions, even if opposite, in relation to the studied vasopeptidases, tests to determine the susceptibility to hydrolysis of both synthetic peptides were carried out. For this, the hypotensins were incubated with the enzymes, in their respective buffers, and later analyzed in an HPLC-C18 system.

**Figure 1:**
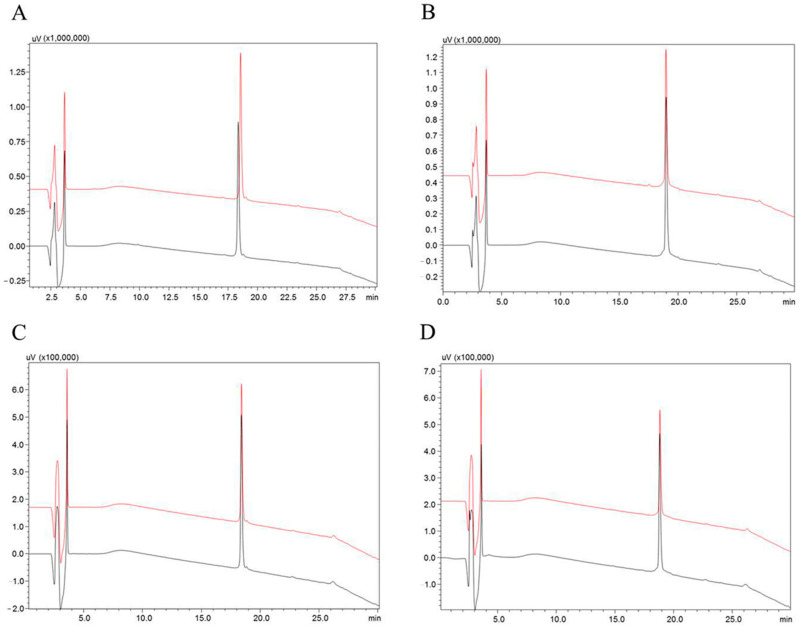
Analysis of the susceptibility to hydrolysis of the hypotensins over the vasopeptidases NEP and ACE. Incubations were carried out in Tris HCl 100 mM, NaCl 50 mM and ZnCl_2_ 10 µM, pH 7.0, for the ACE assays, and, for the NEP assays, in Tris HCl 50 mM, pH 7.5 buffer, for four hours at 37 °C. (**A**) TsHpt-I control (black line) and after incubation with NEP (red line). (**B**) TsHpt-II control (black line) and TsHpt-II incubated with NEP (red line). (**C**) TsHpt-I control (black line) and incubated with ACE (red line). (**D**) TsHpt-II control (black line) and after incubation with ACE (red line). The samples of negative and experimental controls (**A**) TsHpt-I and (**B**) TsHpt-II are shown on the left and right, respectively. The hydrolysis products were considered ≤5%. The gradient used was 20% to 60% solvent B in 20 min, and 60% to 100% solvent B in 5 min (flow 1 mL/min, absorbance at 214 nm). Restek Ultra C-18 5 µm column, 250 × 4.6 mm.

Both hypotensins were resistant to hydrolysis and, therefore, did not behave as substrates for ACE and NEP, even after 4 h of incubation at 37 °C (Figure 1).

Since TsHpt-I demonstrated inhibition of NEP catalytic activity, and was resistant to hydrolysis, kinetic analyses to determine the inhibition constant (Ki) and the mechanism of inhibition of NEP by TsHpt-I were carried out.

**Figure 2:**
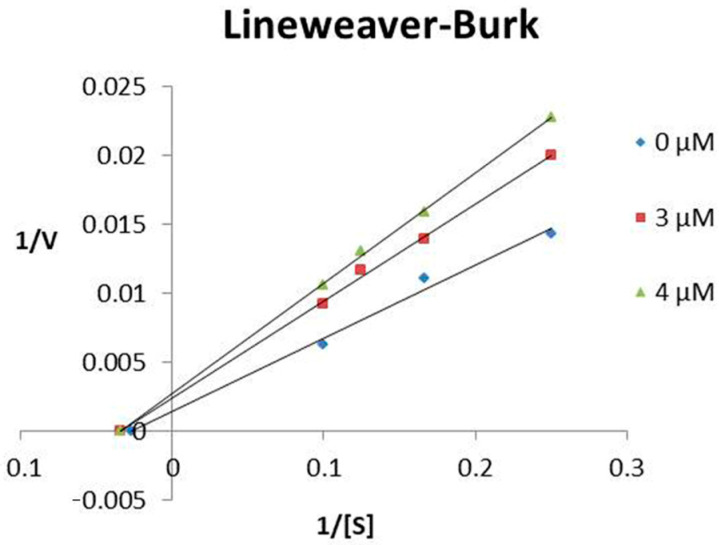
Lineweaver–Burk plot of NEP inhibition by TsHpt-I. The inhibition constant (Ki) was determined using four Abz-RGFK-EDDnp substrate concentrations (4 μM, 6 μM, 8 μM and 10 μM), with variation in the concentration of TsHpt-I (3 μM and 4 μM), keeping the amount of enzyme fixed (1.5 ng). The experiment was performed in triplicate.

TsHpt-I behaved as a non-competitive inhibitor of NEP, presenting an inhibition constant (Ki) of 4.35 μM (Figure 2). The determination of the mechanism and Ki of TsHpt-II on neprilysin activity were not performed as this peptide showed weak interaction with the peptidase (Table 1).

TsHpt-I is the second neprilysin inhibitor from the *Tityus serrulatus* venom described in the literature. Previous observations by our group revealed the presence of a peptide capable of inhibiting human NEP and an NEP-like present in cockroaches, the [des-Arg1]-Proctolin [16]. [des-Arg1]-Proctolin was characterized as a competitive inhibitor of NEP, with an inhibition constant of 0.94 µM [16]. Thus, there may be a synergistic action between these two peptides present in TsV, TsHpt-I and [des-Arg1]-Proctolin, for the inhibition of NEP, and this fact may be related to hypotension associated with scorpionism.

### 2.3. In Vitro Pro-Inflammatory Effects of TsHpt I and -II on Mouse Macrophages

**Figure 3:**
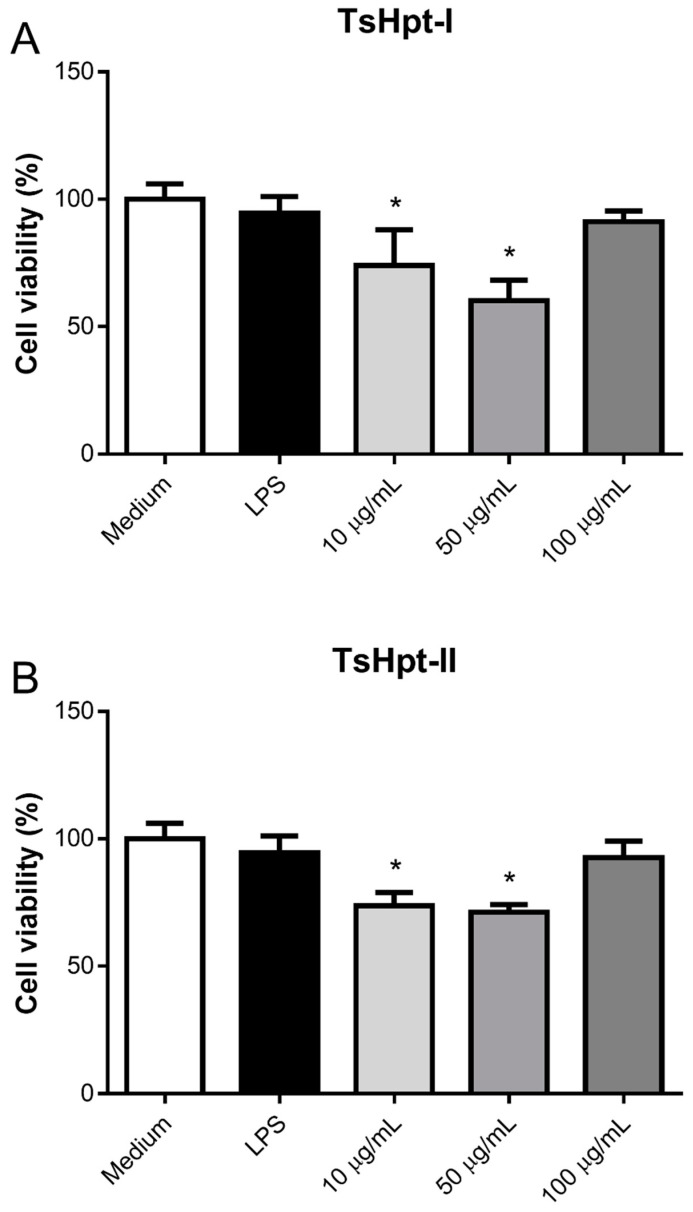
Cytotoxic effect of hypotensins I and II on murine peritoneal macrophages in vitro. Peritoneal exudate macrophages from naïve BALB/c mice were cultured for 24 h in the presence of TsHpt-I (**A**) and -II (**B**) at concentrations of 10 µg/mL, 50 µg/mL and 100 µg/mL. Cells cultured with medium alone or in the presence of 5 µg/mL LPS were used as the controls. Cell viability was determined by the MTT method. The results represent the mean ± SD of two independent experiments performed in triplicate. * *p* < 0.05.

After 24 h of incubation, TsHpt-I and -II exerted intermediate cytotoxicity on naïve murine peritoneal macrophages in vitro, as demonstrated by the statistically significant decrease in cell viability, to about 75% and 60%–70% at concentrations of 10 µg/mL and 50 µg/mL, respectively, when compared to 100% in the control group (medium), as determined by the MTT method (Figure 3). Cytotoxicity was not shown to be dose-dependent (Figure 3).

**Figure 4:**
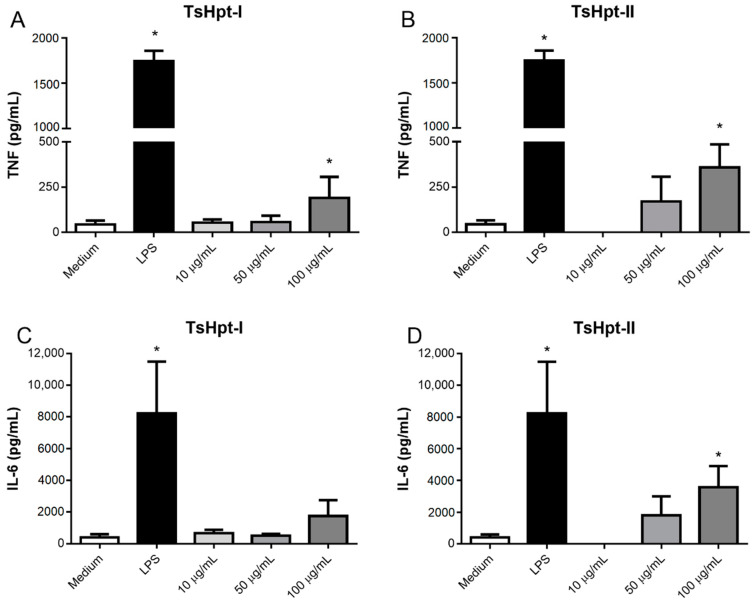
Cytokine production by murine peritoneal macrophages stimulated in vitro with TsHpt-I and -II. Peritoneal exudate macrophages from naïve BALB/c mice were cultured for 24 h in the presence of TsHpt-I (**A**,**C**) and -II (**B**,**D**), at concentrations of 10 µg/mL, 50 µg/mL and 100 µg/mL. Cells cultured with medium alone or in the presence of 5 µg/mL LPS were used as negative and positive controls, respectively. The concentrations of TNF (**A**,**B**) and IL-6 (**C**,**D**) were determined by the CBA method, according to the manufacturer’s instructions. The results represent the mean ± SD of three independent experiments performed in triplicate. * *p* < 0.05.

Despite the moderate cytotoxic effect in the concentrations of 10 µg/mL and 50 µg/mL, TsHpt-I and -II were not able to decrease cellular viability in the higher concentration of 100 µg/mL. Interestingly, in this same concentration, both peptides were able to promote the release of significant levels of TNF, and TsHpt-II also induced the release of IL-6 (Figure 4). None of the peptides were able to induce the release of IL-10, IL-12p70, IFN-γ and MCP-1 (data not shown).

**Figure 5:**
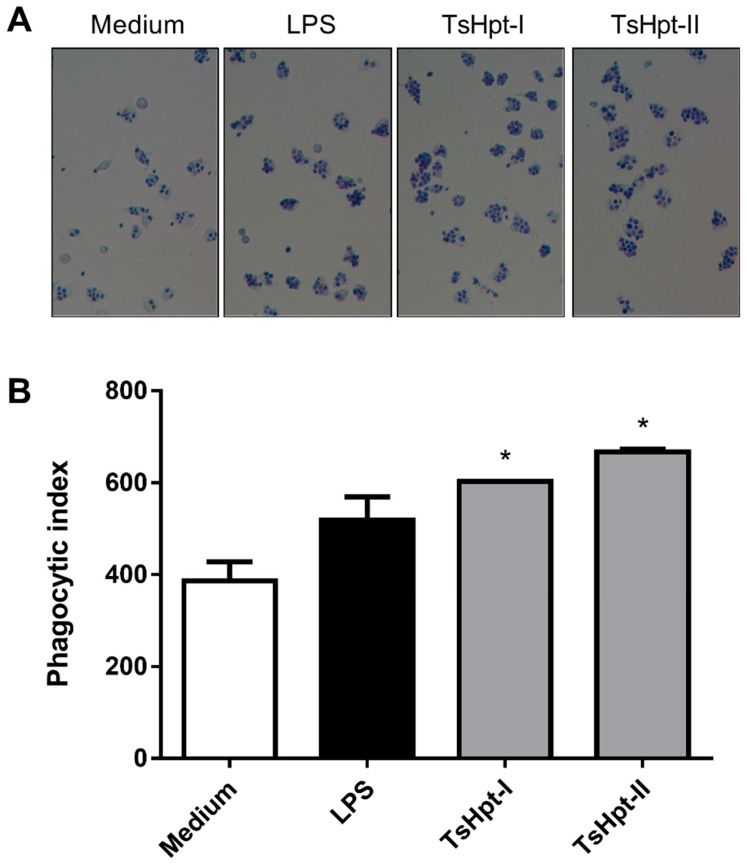
Phagocytic activity of the murine peritoneal macrophages stimulated in vitro with TsHpt-I and -II. Peritoneal exudate macrophages from naïve BALB/c mice were cultured on glass coverslips for 24 h in culture medium or in the presence of LPS (5 μg/mL) or TsHpt-I and -II (100 μg/mL). After this period, the cells were incubated for 1 h with a suspension of *Saccharomyces cerevisiae*. Coverslips were washed, fixed and stained by Giemsa. (**A**) Phagocytosis images in 400× magnification. (**B**) The phagocytic index was calculated based on the percentage of phagocytic cells and on the average number of yeasts per cell, by counting approximately 200 cells per coverslip in 1000× magnification. The results represent one of two reproducible experiments performed in duplicate (mean ± SD). * *p* < 0.05.

In order to confirm the pro-inflammatory effects of both peptides on the biological function of macrophages, the phagocytic activity of macrophages, cultured for 24 h in the presence of TsHpt-I and -II, were analyzed, and compared to the negative and positive controls. Both treatments with TsHpt-I and -II promoted a significant increase in the phagocytic index (Figure 5), demonstrating that the pro-inflammatory action of these peptides also affects the biological function of macrophages.

## 3. Discussion

Identified from the proteomic analyses on the venom of *Tityus serrulatus* (TsV), the hypotensins are peptides made up of 25 amino acid residues that contain two consecutive prolines in their C-terminal portion. Hypotensins are a family of peptides with small structural differences between them, with TsHpt-I being the best-studied member—both the natural molecule and its synthetic counterpart. Tests involving the natural or synthetic TsHpt-I demonstrated that this peptide was able to exert hypotensive activity in normotensive Wistar rats through bradykinin potentiation. The hypothesis about the vasodilation effect is related to NO release, which is a mechanism independent of ACE inhibition [14].

Focusing on clinical conditions related to blood pressure alterations observed in accidents caused by *T. serrulatus*, the present study investigated the possible interaction of TsHpt-I and -II with the metallopeptidases angiotensin converting enzyme (ACE) and neprilysin (NEP). ACE is considered an important molecule in the regulation of blood pressure, as it generates angiotensin II (Ang II) from the cleavage of angiotensin I (Ang I), in addition to degrading bradykinin (Bk) [17]. NEP also acts to control blood pressure through the excretion of Na^+^ and water, as it is capable of degrading natriuretic peptides (ANP, BNP and CNP) [18,19]. Thus, both ACE and NEP are known as vasopeptidases. The third vasopeptidase is the endothelin-converting-enzyme I (ECE-1, EC 3.4.24.71), a metallopeptidase capable of releasing endothelin-I (ET-I), a vasoconstrictor peptide, from the big-endothelin [20].

Regarding TsV, data from the literature describe the presence of an ACE-like one [9,21], capable of converting Ang I to Ang II, and degrading Bk. Hence, the presence of this vasopeptidase in TsV may collaborate with the hypertension observed in accidents involving humans. The possible presence of an NEP-like in TsV has also been described, as well as an inhibitor of this metallopeptidase, called [des-Arg1]-Proctolin [16]. This peptide was characterized as a competitive inhibitor of human NEP, presenting an inhibition constant of 0.94 µM [16]. Proteomic studies also reported the presence of an ECE-like enzyme in TsV [9]. Moreover, high levels of ET-I were observed in the sera of patients after envenomation with the scorpion *Androctonus australis hector*, indicating that molecules of scorpion venoms also have an effect on the endothelin axis. [22]. Therefore, the presence of vasopeptidases and their inhibitors in scorpion venoms may contribute to acute changes caused in the cardiovascular system observed in cases of envenomation [23].

Although the preferred prey of the *Tityus serrulatus* scorpion are insects, such as crickets and cockroaches, its venom is dangerous to humans. According to data from proteomics and transcriptomics studies [9], the toxic effect of TsV in humans may be the result of evolutionarily preserved molecules present in both insects and mammals. This suggestion may explain the presence of ACE-like, ECE-like and NEP-like enzymes in TsV, together with neprilysin inhibitors and hypotensins. [23].

Interestingly, both hypotensins were able to increase the catalytic activity of ACE, but in different ways. While TsHpt-I activated ACE by 64%, TsHpt-II increased by 46% the hydrolysis of the substrate Abz-RGFK-EDDnp. In fact, in studies on the determination of the hypotensive mechanism of TsHpt-I, ACE activation can also be observed; however, the results were not discussed [14]. Studies with NEP also indicated different interactions with hypotensins, and results with TsHpt-I showed that this peptide is a non-competitive inhibitor of NEP, with a Ki value of 4.35 µM. As TsHpt-I is the second NEP inhibitor described in the *T. serrulatus* venom, it is possible that there is a combined action between hypotensin I and [des-Arg^1^]-Proctolin [16], which may be related to the hypotension caused by the envenomation. In contrast, TsHpt-II displayed a low interaction with NEP, and the different results are, probably, the effect of a single difference between the primary structures of the hypotensins. However, future studies of circular dichroism will be needed to clarify this matter.

As hypotensins demonstrated new activities in vitro, cytotoxicity and possible pro- or anti-inflammatory actions were investigated in order to increase our knowledge of these molecules. Both hypotensins have immunomodulatory potential, with pro-inflammatory effects on murine peritoneal macrophages, when used at a concentration of 100 µg/mL. Interestingly, at this concentration, both hypotensins did not exert cytotoxic activity on the tested cells, which makes the two molecules even more interesting, due to their pharmacological potential for the long-term development of new immunostimulants and/or adjuvants. Pucca and collaborators [24] demonstrated the pro-inflammatory effect of three peptides derived from the TsV venom on a strain of murine macrophages, with increased production of IL-6. Similar to the results presented in this work, the effects were subtle compared to LPS, although significant in relation to the negative control. In our study, we observed increased TNF production in the presence of both peptides and increased IL-6 production in the presence of TsHpt-II. As for the mechanism of molecules as hypotensives, it is important to emphasize that both TNF and IL-6 induce vasodilation, and its massive release can even lead to shock.

Cassini-Vieira and colleagues suggested an anti-inflammatory role for TsHpt-I from *T. serrulatus* venom, based on its ability to reduce neutrophil infiltration and TNF production in a murine model of sponge implant-induced inflammation. On the other hand, increased macrophage infiltration was observed in this model, indicating a pro-inflammatory role, which demonstrates the need for further studies on the mechanisms of action of TsHpt-I [25].

Interestingly, both treatments with both peptides promoted a significant increase in the phagocytic index, demonstrating that the pro-inflammatory action of these peptides also affects the macrophages’ biological function. This phenomenon is interesting, considering the possible development of immunomodulators. It is known that adjuvant and/or immunostimulant molecules generally induce a pro-inflammatory environment that favors the activation of antigen-presenting cells and, consequently, the development of adaptive immunity against specific antigens. Increased macrophages’ phagocytic capacity by hypotensins may reflect increased microbicidal activity and/or antigen presentation. The anti-candida and anti-biofilm activities of TistH, a hypotensin present in the venom of the *Tityus stigmurus* scorpion, were recently described and confirmed [26], but functional antigen presentation assays are needed to deepen our knowledge of the immunomodulatory action of these peptides.

Despite the biotechnological potential of hypotensins, the activities already described for these peptides, and the new results showed in the present work, indicate that both molecules do not have a specific target or mechanisms of action. Considering that they are multifunctional toxins present in the *Tityus serrulatus* venom, new studies aiming at drug development should be very carefully carried out in order to minimize unexpected effects.

## 4. Conclusions

Despite the great similarity between the primary structures of hypotensins, different levels of interactions with the vasopeptidases ACE and NEP were observed. Hypotensins increase ACE activity at different levels while TsHpt-I is a non-competitive inhibitor of NEP, suggesting other hypotensive mechanisms for this peptide in addition to those already described. Furthermore, the release of some interleukins may suggest a role for these peptides in the inflammatory response induced by the venom. Hypotensins are multifunctional toxins, and further studies are needed to clarify the potential of these molecules for biotechnological use.

## 5. Material and Methods

### 5.1. *Reagents*

The synthetic peptides TsHpt-I and TsHpt-II were obtained by the solid-phase peptide synthesis method, and purchased from GenOne Biotechnologies (Rio de Janeiro, Brazil). Angiotensin Converting Enzyme (ACE) from rabbit lung, RPMI 1640 medium, LPS from *E. coli* 0127:B8, Trypan Blue, Giemsa stain and glutaraldehyde solution were purchased from Sigma-Aldrich (St. Louis, MO, USA). Neprilysin and the Fluorescence Resonance Energy Transfer (FRET) substrates, Abz-FRK (Dnp) P-OH and Abz-RGFK (Dnp)-OH, were provided by Prof. Adriana Carmona, from the Department of Biophysics of UNIFESP-EPM, São Paulo, SP, Brazil. Acetonitrile and TFA used in RP-HPLC were acquired from J. T. Baker (Avantor, Radnor, PA, USA). Fetal cow serum (FCS) and penicillin and streptomycin antibiotics were purchased from Cultilab (Campinas, SP, Brazil). Tetrazolium salt 3-(4,5-dimethylthiazol-2-yl)-2,5- diphenyltetrazolium bromide (MTT) was purchased from Invitrogen (Waltham, MA, USA). DMSO was purchased from Merck (Darmstadt, Germany). BD Cytometric Bead Array Mouse Inflammation Kit was purchased from BD Biosciences (San Jose, CA, USA). The *Saccharomyces cerevisiae* suspension was obtained from washing and adjusting the concentration of bread yeast (Fleischmann – Petrópolis, RJ, Brazil) in RPMI.

### 5.2. Interactions of Hypotensins with Vasopeptidases ACE and NEP

For ACE assays, 10 µM of each peptide was incubated with 3.0 ng of peptidase and 10 µM of Abz-FRK (Dnp) P-OH substrate, in Tris HCl 100 mM, NaCl 50 mM and ZnCl_2_ 10 µM, pH 7.0 buffer. For NEP, 10 µM of TsHpt-I or -II was incubated with 1.5 ng of peptidase, 3.5 µM Abz-RGFK (Dnp)-OH in Tris HCl 50 mM, pH 7.5 buffer. All reactions occurred at 37 °C, in a final volume of 100 µL, in a Victor 3 fluorimeter (Perkin–Elmer, Waltham, MA, USA) adjusted for excitation and emission readings at 320 and 420 nm, respectively, for 15 min (one reader per minute). Results were obtained in triplicate and analyzed using GraFit 5 (Erithacus software, East Grinstead, West Sussex, UK).

### 5.3. Stability Test of the Synthetic Peptides

The synthetic peptides TsHpt-I and TsHpt-II (30 µM) were incubated with ACE (3.0 ng), in Tris HCl 100 mM, NaCl 50 mM and ZnCl_2_ 10 µM, pH 7.0 buffer, and NEP (1.5 ng), in Tris HCl 50 mM, pH 7.5 buffer, at 37 °C for 4 h. Samples containing only the synthetic peptides were used as the negative control. After incubation, samples were analyzed by reverse phase chromatography on RP-HPLC (Shimadzu, Kyoto, Japan), using a Restek Ultra C-18 column (5 µm, 250 × 4.6 mm). Solvents used were 0.1% TFA in water (solvent A), and acetonitrile plus solvent A (9:1) as solvent B. Separations were performed at a flow rate of 1 mL/min and a 20–60% gradient of solvent B over 20 min. In all cases, elution was followed by the measurement of ultraviolet absorption (214 nm).

### 5.4. Characterization of TsHpt-I as a NEP Inhibitor

To determine the inhibition constant (Ki) of TsHpt-I over NEP, four concentrations of Abz-RGFK (Dnp)-OH (4 µM, 6 µM, 8 µM and 10 µM) and 3 µM and 4 µM of TsHpt-I were tested using 1.5 ng of peptidase in 100 µL of final volume of Tris HCl pH 7.5. The Km value of the substrate used was determined to be 14 µM [27]. Controls without the TsHpt-I were also performed in all assays. The reactions were monitored as described above (item 5.2). The Lineweaver–Burk plot was constructed (1/V × 1/(S)) according to the presented mechanism. The Ki was calculated as described by Segel [28]. All assays were performed in triplicate.

### 5.5. Cell Assays

#### 5.5.1. Murine Peritoneal Macrophages Obtainment

Male young, between 8 and 12 weeks of age and weighing between 20 and 22 g, BALB/c mice adults were used. Mice were obtained from the Central Animal Facility of the Butantan Institute and housed in the Laboratory of Immunochemistry bioterium, Butantan Institute. The mice were kept in boxes lined with shavings, containing 3 animals per box, under natural light, full-time ventilation and exhaustion, filtered water and commercial feed ad libitum. After a period of 2 to 3 days of acclimatization of the animals, they were euthanized in a CO_2_ chamber. All experimental procedures involving animals were in accordance with the ethical principles in animal research adopted by the Brazilian Society of Animal Science and the National Brazilian Legislation no.11.794/08. Animal care and experimental procedures were approved by the Institutional Committee for the Care and Use of Laboratory Animals from Butantan Institute (CEUAIB protocol number 5396310517, approved on 21 June 2017).

Peritoneal exudate cells (BALB/c naïve mice, *n* = 6) were collected by two washes with 5 mL of RPMI medium. The cells obtained were washed twice with RMPI medium, at 400 g, for 10 min, at 18 °C, and resuspended in R10 medium (RPMI + 10% fetal cow serum). After counting in a Neubauer chamber, in the presence of Trypan blue, the cell concentration was adjusted to 2 × 10^6^ cells/mL. Cells were distributed into 96-well culture plates (2 × 10^5^ cells/100 µL/well) and incubated for 2 h at 37 °C and 5% CO_2_. After incubation, non-adherent cells were discarded, and adherent cells (macrophages) were resuspended in R10 medium containing the respective tested stimuli, at different concentrations, in triplicates, in a final volume of 200 µL/well. Cells resuspended in R10 medium were used as a negative control, and cells stimulated with LPS (5 µg/mL) as a positive control. Cells were then incubated for 24 h at 37 °C and 5% CO_2_. After this period, the culture supernatants were collected and stored at −80 °C, for subsequent dosage of cytokines, and cell viability was determined by MTT assay.

#### 5.5.2. Effect of Hypotensins on the Cell Viability and Production of Inflammatory Mediators by Murine Peritoneal Macrophages Stimulated In Vitro with Hypotensins

After a 24-h period of incubation of cells with hypotensins (10 µg/mL, 50 µg/mL and 100 µg/mL), MTT assays were performed, consisting of the addition of 100 µL of R10 containing 0.5 mg/mL of tetrazolium salt 3-(4.5-dimethylthiazol-2-yl)-2,5-diphenyltetrazolium bromide (MTT) in all wells, followed by a new incubation, under the same conditions, for 4 h. Then, the supernatants were discarded, and 100 µL/well of DMSO were added to dissolve the formed crystals. Absorbance was determined in a spectrophotometer at 540 nm. Cell viability was calculated based on the absorbances of the samples and the negative control (cells cultivated only with R10).

The concentration of pro- and anti-inflammatory cytokines (IL-6, IL-10, IL-12p70, IFN-α, MCP-1 and TNF) in the samples incubated with both hypotensins (10 µg/mL, 50 µg/mL and 100 µg/mL) was determined by the CBA method (BD Cytometric Bead Array Mouse Inflammation Kit), according to the manufacturer’s instructions. The samples were acquired in a BD FACSCanto II flow cytometer, and the data were analyzed using BD FCAP Array version 3.0 software.

#### 5.5.3. Effect of TsHpt-I and -II on Phagocytic Function of Murine Peritoneal Macrophages

Peritoneal macrophages from naïve BALB/c mice (*n* = 6) were obtained as described above (item 5.5.1). Cells were distributed in 24-well culture plates (5 × 10^5^ cells/500 µL/well), with glass coverslips inside, and incubated for 2 h at 37 °C and 5% CO_2_. After incubation, non-adherent cells were discarded, and adherent cells (macrophages) were stimulated in vitro with hypotensins, in duplicate, at a concentration of 100 µg/mL, for 24 h at 37 °C and 5% CO_2_. After this period, the culture supernatants were discarded, and the cells were incubated for 1 h with a suspension of *Saccharomyces cerevisiae* at a concentration of 1.5 × 10^6^ yeasts/mL/well. Coverslips were washed 10 times with PBS, fixed with 0.5% glutaraldehyde and stained by Giemsa. Phagocytosis was evaluated by immersion optical microscopy (1000×) and quantified by counting approximately 200 cells per cover slip. The percentage of phagocytic cells and the average number of internalized yeasts per phagocytic cell were calculated. The phagocytic index was obtained by multiplying the two values (percentage × mean) and represents the total phagocytic capacity of each cell population.

### 5.6. Statistical Analysis

The results were statistically analyzed using the GraphPad Prism 5 program, using the one-way ANOVA test followed by Tukey’s post-test. Results with a *p*-value < 0.05 were considered significant.

## Figures and Tables

**Table 1:** Modulations of ACE and NEP activities by interaction with synthetic hypotensins.

	Metallopeptidases			
	ACE		NEP	
	Activation (%)	Inhibition (%)	Activation (%)	Inhibition (%)
TsHpt-I	64	-	-	75
TsHpt-II	44	-	-	11
